# How Normal Cells Can Win the Battle for Survival Against Cancer Cells

**DOI:** 10.1371/journal.pbio.1000423

**Published:** 2010-07-13

**Authors:** William Mair

**Affiliations:** Freelance Science Writer, La Jolla, California, United States of America

[Fig pbio-1000423-g001]During the early stages of tumorigenesis, cancerous cells undergo rapid and uncontrolled cell division as they invade the surrounding tissue. How tumors create space around them to accomplish this invasion is not well understood. A recent study showed that cancerous cells in fruit flies manage this feat by inducing neighboring cells to spontaneously destroy themselves and then filling the vacated space left behind in a process known as cell competition. In this issue of *PLoS Biology*, Yoichiro Tamori et al. provide evidence that this battle also occurs in mammalian tissues and uncover what determines the winners and losers when cells compete.

**Figure pbio-1000423-g001:**
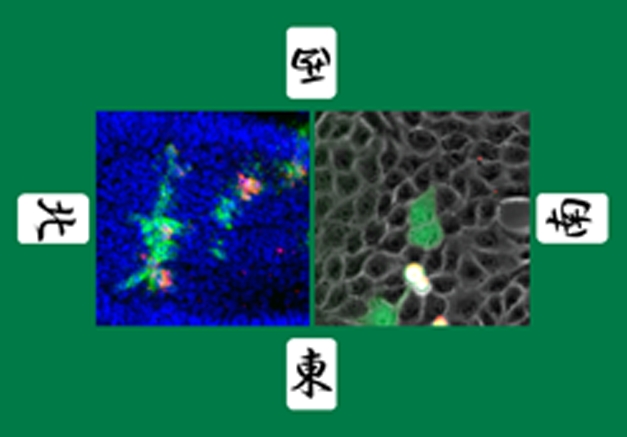
Mahjong-defective cells (green) are out-competed by the adjacent normal cells and become apoptotic (red) both in *Drosophila* imaginal epithelia (left panel) and mammalian cultured epithelial cells (right panel).

Cancers occur when cells undergo a process known as transformation, during which they stop replicating in a controlled manner, leading to unchecked proliferation and tumor formation. The switch from tightly regulated cell division to cancerous growth commonly results from genetic mutations, which are either inherited or acquired through chance mutagenic events or exposure to a carcinogen. Such mutations can turn off genes that usually function as tumor suppressors or alternatively over-activate oncogenes. In both cases, such misregulation leads to cell transformation, uncontrolled growth, and tumors.

When cells begin to over-proliferate in fruit flies, they begin to compete with the healthy ones around them as they fight to clear a path for the invasion of surrounding tissue. The winner of this competition determines whether the tumor will develop or be eliminated. Cells that are genetically modified to overexpress the oncogene *dMyc* out-compete normal cells around them, forcing them to enter “apoptosis,” a programmed self-destruct mechanism usually reserved for damaged cells. However, it is not simply over-proliferation that causes neighboring cells to die, and in some cases the healthy cells fight back. Cells with mutations in the gene “*scribble*” over-proliferate when surrounded by other mutant cells, yet when they are close to normal cells the situation is reversed—the pre-cancerous mutants die and are eliminated from the tissue. Tamori et al. therefore decided to investigate how healthy cells kill nearby transformed cells and whether cell competition might also be occurring in mammalian tissues.

Mutations in a tumor suppressor called lethal giant larvae (Lgl) lead to uncontrolled cell division and tumors. Lgl mutations also lead to cell competition in fruit flies. Because mutations in the mammalian version of Lgl also causes over-proliferation, Tamori et al. suspected that Lgl may play a role in mammalian cancers. However, true cell competition, where one cell dies because of its proximity to another, had not been observed in mammals.

The authors investigated Lgl in both fruit flies and mammals to better understand its role in cell competition. Previous research on the structural properties of the Lgl protein suggested that, rather than acting alone, Lgl was working in tandem with other proteins to exert its effects. To try and identify a potential partner, the authors used a technique called immunoprecipitation to trap both Lgl and any proteins that might be binding to it. They found just one binding partner, which they named Mahjong, noting that winners in this game prevail through “strong competition.”

To determine if deleting Mahjong could induce cell competition in the fruit fly in a manner similar to Lgl, the researchers created fly larvae in which only some cells of the developing wing tissue did not contain the Mahjong gene while the rest did. Initially half of the cells in these mosaic fly tissues lacked Mahjong, but as the tissues developed, the mutant cells began to disappear. A fluorescent probe that identifies cells undergoing apoptosis revealed that cell death was occurring only in mutant cells adjacent to normal cells and not in those surrounded by other Mahjong mutant cells. Therefore, as is the case for Lgl, cells lacking Mahjong are destined to lose in competition with their neighbors.

Having shown a role for Mahjong in cell competition in flies, the researchers then tested if they could induce competition in mammalian cells. To mimic the effects of spontaneous mutations to the Mahjong gene in mammalian tissues, they engineered kidney cells whose copies of the Mahjong gene could be turned off by the addition of the antibiotic tetracycline. Before adding tetracycline, the cells were mixed with normal cells and allowed to grow and form epithelial tissue. Fascinatingly, when tetracycline was then added to the tissue, the cells that lost Mahjong began to die, just as they had in the fly. Their death was self-induced because it did not occur in the presence of a drug that inhibits the apoptotic pathway. In the kidney cells, as in flies, cell suicide was only seen in Mahjong mutants surrounded by normal cells, making this a clear demonstration of cell competition in mammalian tissue.

To further investigate the mechanisms that induce apoptosis in Mahjong- or Lgl-deficient cells, the researchers asked whether they could prevent suicide in cells lacking one gene by overexpressing its partner. In cells without Mahjong, overexpressing Lgl did not prevent apoptosis. The reverse was not true, however, as cells lacking Lgl that would usually self-destruct did not do so when Mahjong was overexpressed. To understand why the mutant cells kill themselves, Tamori et al. tested the levels of the JNK signaling pathway, previously implicated both in cell competition and tumorigenesis. Interestingly, JNK signaling was elevated in both Lgl and Mahjong mutants, and suppressing its activation prevented cell death. Therefore, it is likely that Mahjong and Lgl determine the competitiveness of a cell through their effects on the JNK pathway.

The demonstration of cell competition in mammalian tissues is an exciting step forward into understanding the interactions between carcinogenic cells and their surroundings. These findings identify Mahjong and Lgl as important proteins that can decide whether a cell is destined to survive that competition. Influencing who wins when cells compete may yield novel therapies for treatment of human cancers if researchers can develop interventions to swing the balance of power back toward normal cells.

**Tamori Y, Bialucha CU, Tian A-G, Kajita K, Huang Y-C (2010) Involvement of Lgl and Mahjong/VprBP in Cell Competition. doi:10.1371/journal.pbio.1000422**

